# Lactopeptine

**Published:** 1878-08

**Authors:** 


LACTOPEPTINE.—Pepsin is unquestionably a valuable remedy in some cases of indigestion, but does not seem to meet all the requirements of many dyspeptic cases. Lactopeptine is presented to the profession as meeting all the indications in cases of mal-nutrition and non-assimilation, composed according to the formula, of Ptyalin, Pepsin, Pancreatine Hydrochloric and Lactic Acids. It is claimed to be a combination of all the digestive agents. If we can prescribe chemically for disorder of the digestive function, such a combination would appear worthy of a trial, and experience has demonstrated its value in many cases. Dr. Merritt remarks: "The more my experience in its varied applicability extends, the more its beneficial effects appear."—*Buffalo Medical and Surgical Journal, Dec.* 1877.

